# Insights into the chloroplast genome diversity of the genus *Isatis* in China

**DOI:** 10.1186/s12870-026-08240-3

**Published:** 2026-01-26

**Authors:** Min Wei, Chengxiang Wang, Yong Su, Hongzhuan Shi, Liangju Ma, Tao Bao, Qiaosheng Guo

**Affiliations:** 1 Institute of Chinese Medicinal Materials, Nanjing Agricultural University, Nanjing City, Jiangsu Province 210095 P. R. China; 2 China Resources Sanjiu Medical & Pharmaceutical Co., Ltd, Shenzhen City, 518000 P. R. China; 3 Shenzhen Traditional Chinese Medicine Manufacturing Innovation Center Co., Ltd., Shenzhen City, 518110 P. R. China; 4 Key Laboratory of Horticultural Crop Germplasm Innovation and Utilization (Coconstruction by Ministry and Province), Institute of Horticulture, Anhui Academy of Agricultural Sciences, Hefei, 230001 China

**Keywords:** Isatis, Chloroplast genome, Structural characteristics, Phylogeny, Molecular marker

## Abstract

**Background:**

The genus *Isatis* contains medicinally important but taxonomically controversial species in China. Reliable genomic resources are urgently needed for accurate species identification and phylogenetic clarification.

**Results:**

We assembled and characterized the complete chloroplast genomes of seven *Isatis* species. The genomes (153,260–153,872 bp) exhibited the typical quadripartite structure. Sequence variation was heterogeneous: the small single-copy (SSC) region was the most polymorphic (12.3 single-nucleotide polymorphisms (SNPs)/kb), followed by the large single-copy (LSC, 8.9 SNPs/kb) and inverted repeat (IR, 1.7 SNPs/kb) regions. Noncoding sequences showed 3.2-fold greater polymorphism than coding sequences. The *rpl32–trn*L intergenic spacer was identified as a promising molecular marker due to its high nucleotide diversity (*π* = 0.0582). While most protein-coding genes were under strong purifying selection, *ycf1* and *ycf2* exhibited elevated substitution rates. Phylogenomic analyses strongly supported the monophyly of the tribe Isatideae and the genus *Isatis* (100% bootstrap support (BS)), validating the recent inclusion of *I. gymnocarpa* and *I. multicaulis*. We detected significant misidentification in public databases for key taxa (*I. costata*, *I. tinctoria*, *I. indigotica*). The *rpl32–trn*L spacer effectively distinguished most species but revealed the non-monophyly of *I. costata*, with one lineage being indistinguishable from *I. tinctoria*. The phylogenetic separation of the morphologically similar *I. minima* and *I. violascens* suggests potential convergent evolution.

**Conclusion:**

Our study provides essential chloroplast genomic resources for *Isatis*. The findings not only clarify long-standing taxonomic controversies and validate recent reclassifications but also highlight the utility of the *rpl32–trn*L spacer as a powerful marker for species discrimination and evolutionary studies within this genus.

**Supplementary Information:**

The online version contains supplementary material available at 10.1186/s12870-026-08240-3.

## Introduction

The genus *Isatis* Tourn. ex L. comprises a group of plants within the family Brassicaceae Burnett that hold significant medicinal and economic value [1]. Widely distributed across Eurasia and the Mediterranean region, several species—such as *Isatis cappadocica* Desv. and *Isatis tinctoria* L.—serve as natural resources for the dye [2], cosmetics [3, 4], and pharmaceutical industries [5, 6]. However, long-standing controversies regarding the taxonomy and phylogenetic relationships within this genus have considerably hindered the accurate identification and quality control of medicinal materials. First, inconsistencies exist among authoritative taxonomic references: of the six species recorded in the *Flora of China* [7], two, *Isatis multicaulis* (Kar. & Kir.) Jafri and *Isatis gymnocarpa* (Fisch. ex DC.) Al-Shehbaz, Moazzeni & Mumm., were transferred from the neighbouring genera *Pachypterygium* Bunge and *Tauscheria* Fisch. ex DC., respectively. In contrast, the Chinese Pharmacopoeia recognizes only *Isatis indigotica* Fortune ex Lindl. as the official source of “Isatidis Radix” [8], whereas major taxonomic treatments such as the *Flora of China* regard *I. indigotica* as a synonym of *I. tinctoria* L [7]. This taxonomic controversy, combined with the high morphological similarity and overlapping distributions of many *Isatis* species, poses considerable challenges for traditional identification methods. Although attempts have been made to develop chloroplast mini-barcodes for distinguishing *I. indigotica* from *I. tinctoria*, their applicability and effectiveness across a broader range of *Isatis* species remain to be thoroughly evaluated [9, 10].

Resolving these taxonomic controversies requires a well-characterized phylogenetic framework for *Isatis*. In recent years, a new classification system has divided the subfamily Brassicoideae into several supertribes, with the tribe Isatideae placed within the supertribe Brassicodae [11–13]. Nevertheless, the evolutionary position of *Isatis* within this system and the validity of previously proposed genus transfers based on morphology and nuclear genes (e.g., *I. multicaulis* and *I. gymnocarpa*) still require verification using independent data such as chloroplast genomes [14, 15]. Moreover, existing studies on interspecific relationships within the genus—particularly among morphologically and ecologically similar species such as *Isatis minima* Bunge and *Isatis violascens* Bunge—have relied largely on single-marker approaches [16, 17]. These studies provide limited phylogenetic information, making it difficult to discern whether these phenotypic similarities are due to close relationships or convergent evolution.

Given its compact structure, sequence conservation, and high information content, the chloroplast genome has emerged as a powerful tool for resolving phylogenetic relationships among closely related taxa and for developing highly discriminatory molecular markers [18–20]. To systematically address the aforementioned issues in the taxonomy and phylogeny of *Isatis*, this study employs Illumina high-throughput sequencing to sequence and compare the chloroplast genomes of seven representative species: *I. tinctoria*, *Isatis costata* C.A. Mey., *I. minima*, *I. violascens*, *I. multicaulis*, *I. gymnocarpa*, and *I. indigotica*. The aims are to assess and develop new chloroplast DNA barcodes suitable for species identification across the entire genus, to clarify the phylogenetic placement and monophyly of *Isatis* within the higher-level classification of Brassicaceae, and to evaluate the consistency of previous genus-level reclassifications based on nuclear data with chloroplast genome evidence. These findings provide a solid genomic foundation for the conservation of *Isatis* resources, accurate identification of medicinal materials, and further study of the evolutionary history of this genus.

## Materials and methods

### Plant materials

**Fig. 1:**
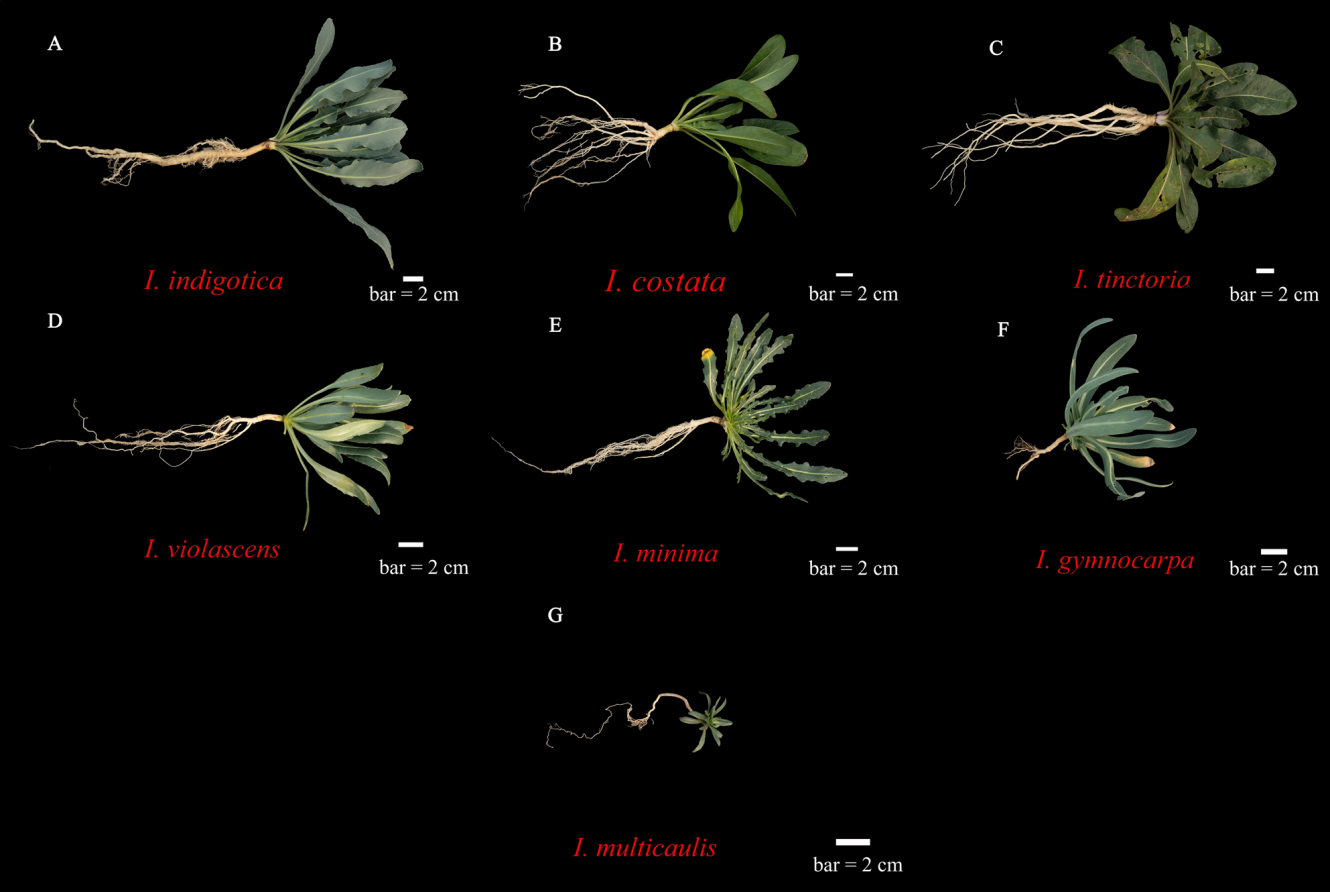
Morphological characteristics of seven *Isatis* species. The samples are labeled as follows: **A** *I. indigotica*, **B** *I. costata*, **C** *I. tinctoria*, **D** *I. violascens*, **E** *I. minima*, **F** *I. gymnocarpa*, **G** *I. multicaulis*. Scale bar: 2 cm (applicable to **A**-**G**)

The plant materials utilized in this study are comprehensively summarized and illustrated in Fig. 1. A total of seven *Isatis* species were collected from their natural habitats. Specifically, *I. indigotica* was sampled from Huangshi City, Hubei Province, while the other six species (*I. tinctoria*, *I. costata*, *I. minima*, *I. violascens*, *I. multicaulis*, and *I. gymnocarpa*) were collected from various locations within the Xinjiang Uygur Autonomous Region, China. All the collected specimens were authenticated by Professor Qiaosheng Guo of Nanjing Agricultural University. Voucher specimens for each of the seven species are preserved in the research collection of the Institute of Chinese Medicinal Materials at Nanjing Agricultural University, Nanjing, China. For each sample, healthy young leaves were selected and rapidly dried in silica gel until DNA extraction. Detailed collection information, including geographical coordinates, is provided in Table S1.

To construct a robust phylogenetic framework, we supplemented our data with all available complete chloroplast genome sequences of *Isatis* and related taxa retrieved from the NCBI Organelle Genome Database [4, 9, 21]. The final dataset encompasses sequences from congeneric species, representatives from closely related genera within the tribe Isatideae, and outgroup species from other tribes of Brassicaceae. The accession numbers, sources, and corresponding references for all the downloaded sequences are also listed in Table S1.

### DNA extraction, sequencing, assembly, and annotation

Genomic DNA was extracted from leaf tissues using a Plant Genomic DNA Extraction Kit (Dp360; Tiangen Biotech, Beijing, China). High-throughput sequencing was performed on the BGISEQ-T7 platform (BGI-Shenzhen, China), generating approximately 7.1–9.0 Gb of raw data per sample. The raw reads were subsequently used for chloroplast genome assembly without prior quality filtering, as is standard practice for organelle genome assembly due to its uneven base composition [22]. The quality of the raw data was high, with Q20 and Q30 scores ranging from 95.7 to 98.5% and 89.3–95.9%, respectively.

The chloroplast genomes were assembled de novo using GetOrganelle (v1.7.7.1) [23] with the -F embplant_pt parameter. The assembly quality was rigorously assessed by (1) confirming the complete circularization of the genome; (2) verifying the presence of the typical quadripartite structure, consisting of a large single-copy (LSC), a small single-copy (SSC), and two inverted repeat (IR) regions; and (3) leveraging the inherent high coverage depth resulting from the high copy number of chloroplast DNA.

Annotation was performed using a dual-strategy approach: (1) structural annotation with GeSeq to identify IR regions and determine genome orientation [24] and (2) functional annotation with CPGAVAS2 for gene prediction [25]. Manual curation was subsequently applied to refine gene boundaries and intron/exon structures, and genome maps were generated using OGDRAW (v1.3.1) [26]. The complete chloroplast genome sequences were submitted to the NCBI database, and GenBank accession numbers were obtained.

### Comparative genomic analysis and identification of divergent hotspots

The chloroplast genomes of seven *Isatis* species were compared using the mVISTA online tool (https://genome.lbl.gov/vista/mvista/) in Shuffle-LAGAN mode, with the *I. multicaulis* (GenBank: PQ059879) chloroplast genome serving as the reference. The expansion and contraction of the IR and SC region boundaries were visualized using IRscope (https://irscope.shinyapps.io/irapp/).

To identify mutation hotspots, the nucleotide diversity (π) across the aligned chloroplast genomes was calculated using DnaSP software (v6.12.03) [27]. The analysis was performed with a sliding window of 400 bp and a step size of 200 bp, using the *I. multicaulis* genome as the alignment reference. The region with the highest π value, the *rpl32–trn*L intergenic spacer, was identified as the most divergent hotspot.

### Repeat sequence analysis

Simple sequence repeats (SSRs) were identified using the MISA tool (https://webblast.ipk-gatersleben.de/misa/), with minimum repeat thresholds set to 8, 5, 4, 3, 3, and 3 for mono-, di-, tri-, tetra-, penta-, and hexanucleotides, respectively, and a minimum distance of 100 bp between adjacent SSRs. Dispersed repeats were detected using REPuter (https://bibiserv.cebitec.uni-bielefeld.de/reputer/manual.html) [28], with the following parameters: maximum number of repeats = 50; minimum repeat length = 30; Hamming distance = 3; and four repeat types, including forward (f), reverse (r), complement (c), and palindromic (p).

### Codon usage bias and selective pressure analysis

Relative synonymous codon usage (RSCU) was calculated for each sample using DAMBE software (v7.0.35), with redundant genes excluded and non-ATG start codons corrected during amino acid editing [29]. To investigate the patterns of natural selection, we determined the pairwise Ka/Ks ratios for all coding sequences (CDSs) using TBtools (v2.056). This analysis allowed for an assessment of selective pressure across the *Isatis* sample set [30].

### Phylogenetic analysis

To ensure robust phylogenetic inference, we analysed 26 *Isatis* chloroplast genomes, incorporating all publicly available sequences from NCBI along with seven newly sequenced accessions. *Sisymbrium altissimum* L. (Sisymbrieae, Brassicodae) was selected as the outgroup for stable rooting. The ingroup consisted of 26 *Isatis* sequences to resolve infrageneric relationships, supplemented by three representatives from related Isatideae genera (*Myagrum perfoliatum* L., *Schimpera arabica* Hochst. & Steud., and *Conringia planisiliqua* Fisch. & C. A. Mey. (a synonym of *Iljinskaea planisiliqua* (Fisch. & C.A.Mey.) Al-Shehbaz, Özüdoğru & D.A. German)) to test monophyly. *Goldbachia laevigata* (M. Bieb.) DC. (Calepineae) was included as a contextual reference within supertribe Brassicodae [13].

Phylogenetic analyses were conducted using both maximum likelihood (ML) and Bayesian inference (BI) methods. The ML analysis was performed with IQ-TREE 2 (version 2.4.0; https://github.com/iqtree/iqtree2) [31], with the best-fit substitution model (TVM + F + I + R4) selected automatically according to the Bayesian information criterion and branch support assessed with 1000 bootstrap replicates [32]. The BI analysis was performed using MrBayes (version 3.2.7a; http://mrbayes.sourceforge.net/) under the GTR + Γ model of sequence evolution. Two independent runs of four Markov chain Monte Carlo (MCMC) simulations each were conducted for 200,000 generations, sampling every 500 generations. The first 25% of the trees were discarded as burn-in. The phylogenetic trees were visualized and annotated using the Interactive Tree of Life (iTOL) (accessed on [29 Sep. 2025]; https://itol.embl.de/) [33].

### Analysis of sequence divergence in *rpl*32–*trn*L

To validate the utility of the identified *rpl32–trn*L hotspot for species discrimination, we employed a previously published primer pair (Forward: 5’-ACCTTGATGCAATAATAAACAAAGA-3’; Reverse: 5’-AAAATGAAAACTTCTCCAAAATGC-3’) [9]. These primers were used to amplify and sequence the region from 40 samples (Table S2). PCR amplification was performed in a 50 µL reaction mixture containing 47 µL of Tsingke Golden Mix, 1 µL each of 10 µM forward and reverse primers, and 1 µL of genomic DNA. The thermal cycling protocol consisted of an initial denaturation at 98 °C for 2 min; 35 cycles of denaturation at 98 °C for 10 s, annealing at 54 °C for 10 s, and extension at 72 °C for 10 s; followed by a final extension at 72 °C for 5 min. The PCR amplicons were sequenced bidirectionally on an AB 3730Xl DNA Sequencer (Applied Biosystems, U.S.A.) by Tsingke Biological Co., Ltd.

To root the phylogenetic trees, the *rpl32–trn*L intergenic spacer sequences of two closely related species, *Myagrum perfoliatum* L. (GenBank: JQ911317.1) from the tribe Isatideae and *Sisymbrium orientale* L. (GenBank: JQ911343.1) from the tribe Sisymbrieae, were retrieved from NCBI and included in the alignment as outgroups. The raw bidirectional sequencing reads were assembled and base-called using CExpress to generate consensus sequences for each sample. All 40 consensus sequences of the *rpl32–trn*L intergenic spacer and 2 outgroups were aligned using the ClustalW algorithm implemented in MEGA version 11.0.13 with default parameters. The alignment was manually checked and trimmed.

Phylogenetic trees were reconstructed from this alignment using both ML and Neighbor-Joining (NJ) methods in MEGA 11.0.13. For the ML analysis, the Kimura 2-parameter model was employed, with rate heterogeneity among sites modeled using a discrete Gamma distribution (+ G, 5 categories). Branch support was assessed from 1,000 bootstrap replicates. For the NJ tree, pairwise distances were computed using the Maximum Composite Likelihood method under a uniform rates model, with gaps/missing data handled via pairwise deletion. Node support was also evaluated by 1,000 bootstrap replicates.

## Results

### General characteristics of the *Isatis* chloroplast genomes

**Fig. 2:**
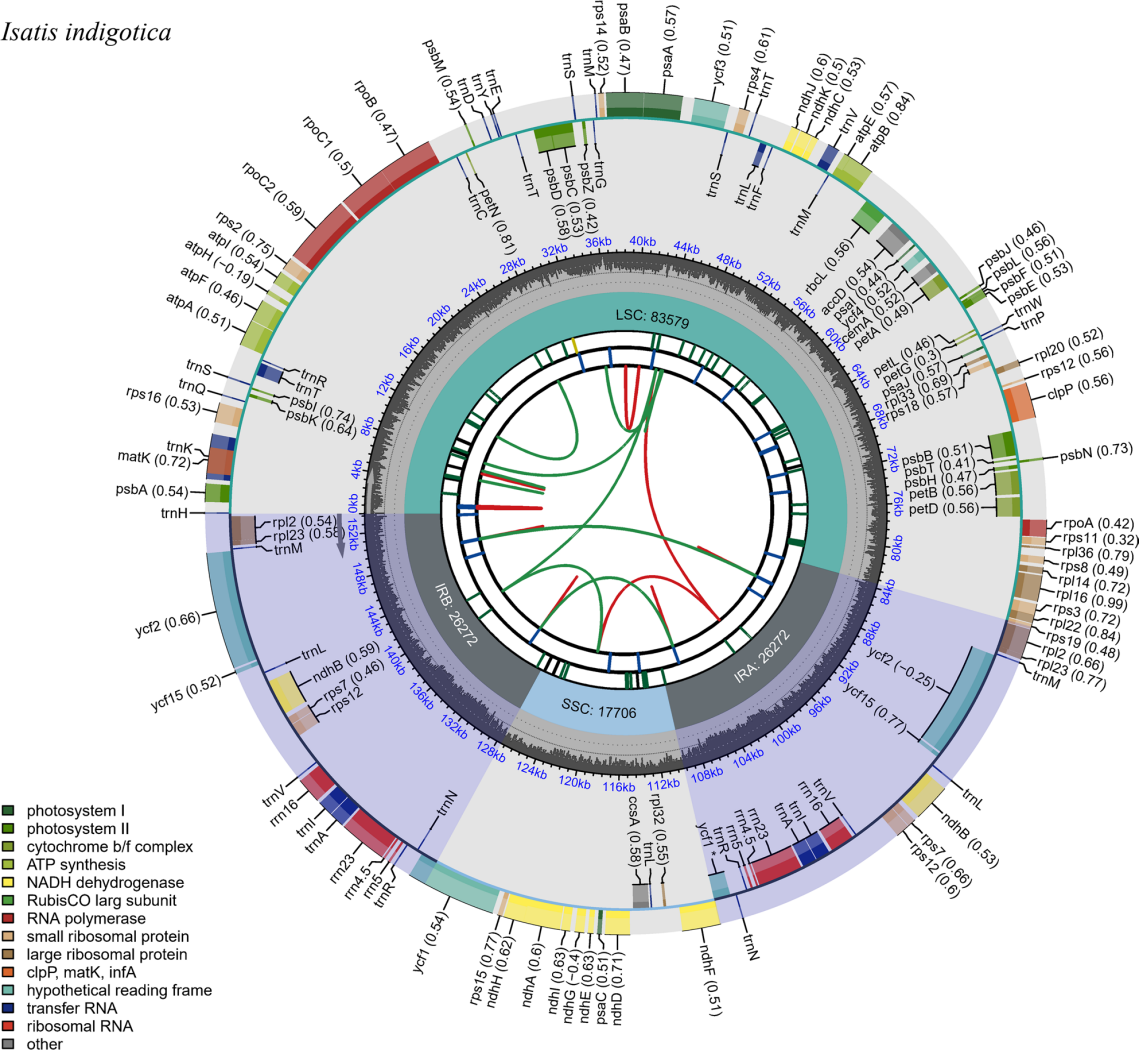
Circular chloroplast genome map of *Isatis* species. The diagram comprises six concentric rings, each depicting distinct genomic features. From the innermost to outermost layers, these include spatially distributed repeats (forward orientation in red, reverse in green), tandem repeats (blue bars), short tandem repeats (green bars), the quadripartite organization (LSC, SSC, and paired IR regions labeled IRA/IRB) with annotated lengths, GC content gradients, and color-coded gene annotations. Numeric values in parentheses adjacent to gene labels reflect codon usage bias

The complete chloroplast genomes of seven *Isatis* species were successfully assembled, and their circular structures are illustrated in Fig. 2. The genome lengths ranged from 153,260 bp (*I. violascens*) to 153,872 bp *(I. costata*), with highly conserved GC contents ranging from 36.46 to 36.52%. Further comparison revealed that the maximum difference in length among the genomes was 612 bp, whereas the minimum difference was only 7 bp.

Gene annotation revealed four functional categories: self-replication, photosynthesis, uncharacterized genes, and others (Table 1). Intron analysis revealed 17 genes with splicing elements: 15 single-intron genes (*rpl2*,* rpl16*,* rps16*,* rpoC1*,* trnA-UGC*,* trnE-UCC*,* trnK-UUU*,* trnL-UAA*,* trnV-UAC*,* trnT-UGU*,* ndhA*,* ndhB*,* petB*,* petD*, and *atpF*) and two double-intron genes (*clpP* and *ycf3*).

**Table 1:** Gene composition of the Chloroplast genomes in the genus *Isatis*

Category	Gene group	Gene name
Photosynthesis	Subunits of photosystem I	*psa*A, *psa*B, *psa*C, *psa*I, *psa*J
	Subunits of photosystem II	*psb*A, *psb*B, *psb*C, *psb*D, *psb*E, *psb*F, *psb*H, *psb*I, *psb*J, *psb*K, *psb*L, *psb*M, *psb*N, *psb*T, *psb*Z
	Subunits of NADH dehydrogenase	*ndh*A*, *ndh*B*(2), *ndh*C, *ndh*D, *ndh*E, *ndh*F, *ndh*G, *ndh*H, *ndh*I, *ndh*J, *ndh*K
	Subunits of cytochrome b/f complex	*pet*A, *pet*B*, *pet*D*, *pet*G, *pet*L, *pet*N
	Subunits of ATP synthase	*atp*A, *atp*B, *atp*E, *atp*F*, *atp*H, *atp*I
	Large subunit of rubisco	*rbc*L
	Subunits photochlorophyllide reductase	-
Self-replication	Proteins of large ribosomal subunit	*rpl*14, *rpl*16*, *rpl*2*(2), *rpl*20, *rpl*22, *rpl*23(2), *rpl*32, *rpl*33, *rpl*36
	Proteins of small ribosomal subunit	*rps*11, *rps*12**(2), *rps*14, *rps*15, *rps*16*, *rps*18, *rps*19, *rps*2, *rps*3, *rps*4, *rps*7(2), *rps*8
	Subunits of RNA polymerase	*rpo*A, *rpo*B, *rpo*C1*, *rpo*C2
	Ribosomal RNAs	*rrn*16S(2), *rrn*23S(2), *rrn*4.5(2), *rrn*5S(2)
	Transfer RNAs	*trn*A-UGC*(2), *trn*C-GCA, *trn*D-GUC, *trn*E, *trn*F-GAA, *trn*G-GCC, *trn*H-GUG, *trn*I*(2), *trn*K-UUU*, *trn*L-CAA(2), *trn*L-UAA*, *trn*L-UAG, *trn*M-CAU(4), *trn*N-GUU(2), *trn*P-UGG, *trn*Q-UUG, *trn*R-ACG(2), *trn*R-UCU, *trn*S-GCU, *trn*S-GGA, *trn*S-UGA, *trn*T-CGU*, *trn*T-GGU, *trn*T-UGU, *trn*V-GAC(2), *trn*V-UAC*, *trn*W-CCA, *trn*Y-GUA
Other genes	Maturase	*mat*K
	Protease	*clp*P**
	Envelope membrane protein	*cem*A
	Acetyl-CoA carboxylase	*acc*D
	c-type cytochrome synthesis gene	*ccs*A
	Translation initiation factor	-
	Other	-
Genes of unknown function	Conserved hypothetical chloroplast ORF	#*ycf*1, *ycf*1, *ycf*15(2), *ycf*2(2), *ycf*3**, *ycf*4

Gene*: Gene with one intron; Gene**: Gene with two introns; #Gene: Pseudogene; Gene (2): Number of copies of multicopy genes

### Comparative analysis of chloroplast genomes in *Isatis*

The size of the LSC region varies from 83,181 bp (*I. violascens*) to 83,661 bp (*I. costata*), with a maximum difference of 480 bp and a minimum difference of 16 bp. Additionally, the size of the SSC region ranged from 17,604 bp (*I. gymnocarpa*) to 17,717 bp (*I. tinctoria*), with a maximum difference of 113 bp and a minimum difference of 3 bp. The IR regions varied in size from 26,224 bp (*I. gymnocarpa*) to 26,283 bp (*I. tinctoria*), with a maximum difference of 59 bp and a minimum difference of 4 bp. A total of 132 genes were identified, including 37 tRNAs, 8 rRNAs, and 87 protein-coding genes (PCGs). Among these, 14 tRNAs and 8 rRNAs are located within the IR regions, as detailed in Table 2.

**Fig. 3:**
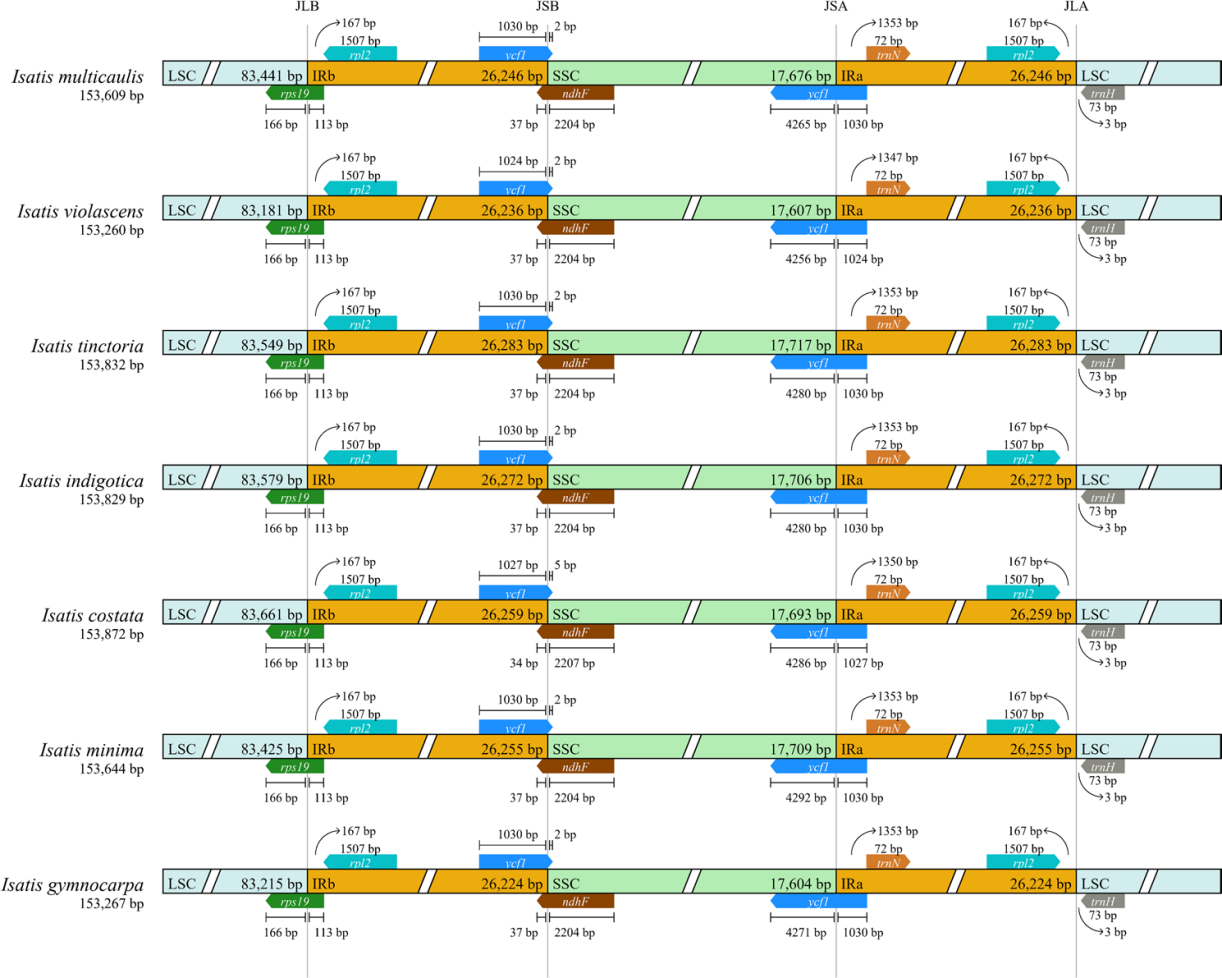
Comparison of the junction sites between the single-copy and IR regions in *Isatis* chloroplast genomes. The expansion and contraction of the IR boundaries are shown

Analysis of the inverted repeat sequences revealed that the rps19 gene is located at the junction of the LSC and IRb regions. The ndhF and #*ycf1* genes are located at the boundary of IRa and SSC, with the majority of the ndhF gene extending into the SSC region. The *ycf1* gene is positioned at the junction of SSC and IRb, predominantly extending into the SSC region. The *trn*H gene is located at the junction of IRa and LSC, with a distance of 3 bp, whereas the *rpl2* gene slightly contracts towards the IRa region. As shown in Fig. 3, the IR/SC junctions are nearly identical in length, with only minor expansions and contractions, indicating a high degree of similarity among the chloroplast genomes of the seven *Isatis* species.

**Fig. 4:**
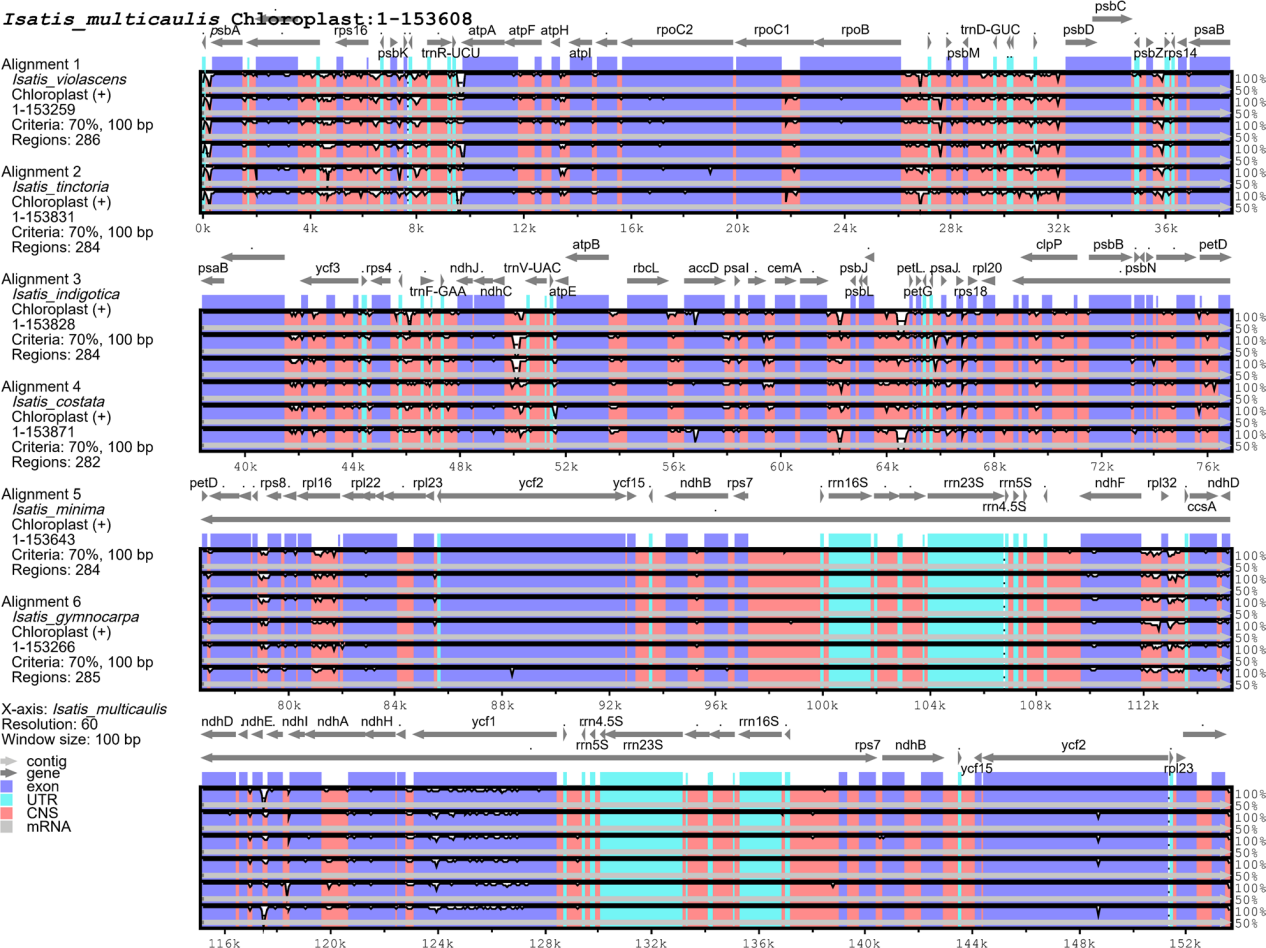
Whole-genome alignment and sequence divergence analysis of the seven *Isatis* chloroplast genomes. The chloroplast genome of *I. multicaulis* was used as the reference. The x-axis represents the genome coordinates, and the y-axis shows the percent identity (50–100%) of the aligned regions to the reference

The overall sequences of the chloroplast genomes of the seven *Isatis* species were mapped using mVISTA, with the annotation of *I. gymnocarpa* serving as the reference. These seven species demonstrate a high level of sequence similarity, with the IR regions being more conserved than the single-copy regions. Although coding regions generally exhibited higher conservation than noncoding regions did, we identified four genes with relatively high levels of variation as hypervariable regions: *ycf1* (located at the SSC-IRA boundary), *ycf2*, *ndhF*, and *rpoC2*. This phenomenon may be related to the functional specificity of these genes and relaxed selective constraints. Furthermore, significant divergence was observed in noncoding intergenic spacers (IGSs), including highly variable regions near the *trn*F-GAA, *trn*V-UAC, *pet*D, and *rpl*16 loci, as depicted in Fig. 4.

**Table 2:** Summary of the Chloroplast genome characteristics of 7 species of the *Isatis* genus

Species	Size (bp)	GenBank Accession No.	GC Content (%)	LSC Length (bp)	SSC Length (bp)	IR Length (bp)	Gene Number	Protein Coding Gene Number	rRNA Gene Number	tRNA Gene Number
*I. indigotica*	153,829	PQ158474	36.47	83,579	17,706	26,272	132	87	8	37
*I. costata*	153,872	PQ158475	36.51	83,661	17,693	26,259	132	87	8	37
*I. minima*	153,644	PQ093985	36.46	83,425	17,709	26,255	132	87	8	37
*I. violascens*	153,260	PQ158472	36.50	83,181	17,607	26,236	132	87	8	37
*I. gymnocarpa*	153,267	PQ093986	36.52	83,215	17,604	26,224	132	87	8	37
*I.* multicaulis	153,609	PQ059879	36.49	83,441	17,676	26,246	132	87	8	37
*I. tinctoria*	153,832	PQ158473	36.47	83,549	17,717	26,283	132	87	8	37

### SSR analysis of Chloroplast genomes in *Isatis*

**Fig. 5:**
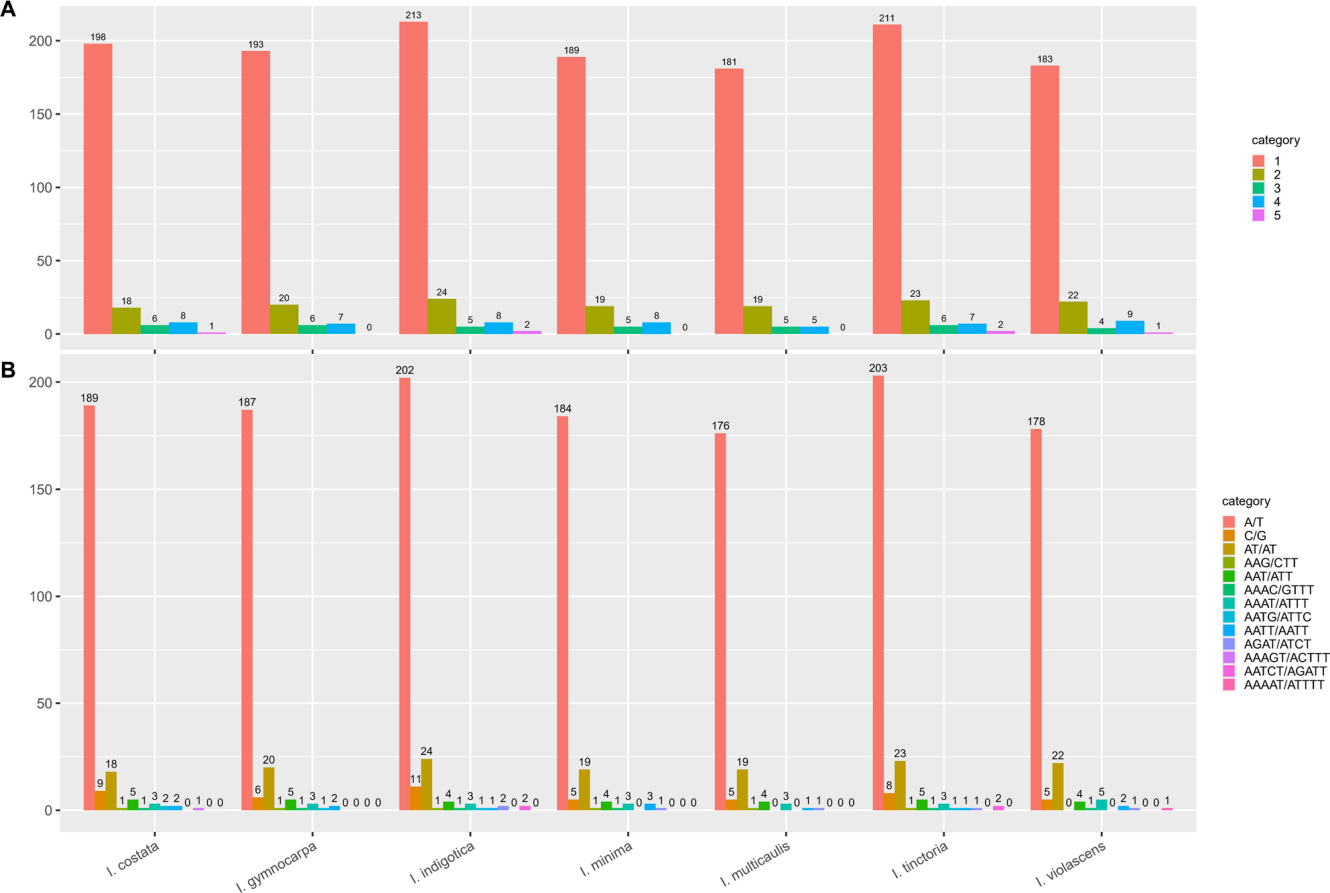
Analysis of SSRs in *Isatis* chloroplast genomes. **A** Number of different SSR types. **B** Distribution of SSRs across LSC, SSC, and IR regions

In this study, MISA software was used to detect SSRs within the chloroplast genomes of *Isatis* species, and the results are detailed in Fig. 5. Analysis of SSRs in the chloroplast genomes of seven *Isatis* species revealed that the total number of SSRs ranged from 210 (*I. multicaulis*) to 252 (*I. indigotica*), as shown in Table 3. Mononucleotide repeats were the most abundant type across all species (ranging from 181 to 213), significantly outnumbering other repeat types. The numbers of dinucleotide, trinucleotide, and tetranucleotide repeats were relatively low and comparable, whereas pentanucleotide repeats were the rarest and were detected only in *I. costata*, *I. indigotica*, *I. violascens*, and *I. tinctoria*. Furthermore, the predominant A/T repeats were highly abundant (176 to 203), further highlighting a strong base composition bias in the SSR motifs. Notably, the A/T-only pentanucleotide repeat AAAAT/ATTTT was specifically found only in *I. violascens*, as detailed in the complete SSR dataset (Supplementary Material 3).

**Table 3:** Distribution of SSRs in the Chloroplast genomes of seven *Isatis* species

Species	Total	Mononucleotide	Dinucleotide	Trinucleotide	Tetranucleotide	Pentanucleotide	A/T Repeats
*I. costata*	231	198	18	6	8	1	189
*I. gymnocarpa*	226	193	20	6	7	0	187
*I. indigotica*	252	213	24	5	8	2	202
*I. minima*	221	189	19	5	8	0	184
*I. multicaulis*	210	181	19	5	5	0	176
*I. violascens*	219	183	22	4	9	1	178
*I. tinctoria*	249	211	23	6	7	2	203

### Nucleotide diversity analysis of chloroplast genomes in *Isatis*

**Fig. 6:**
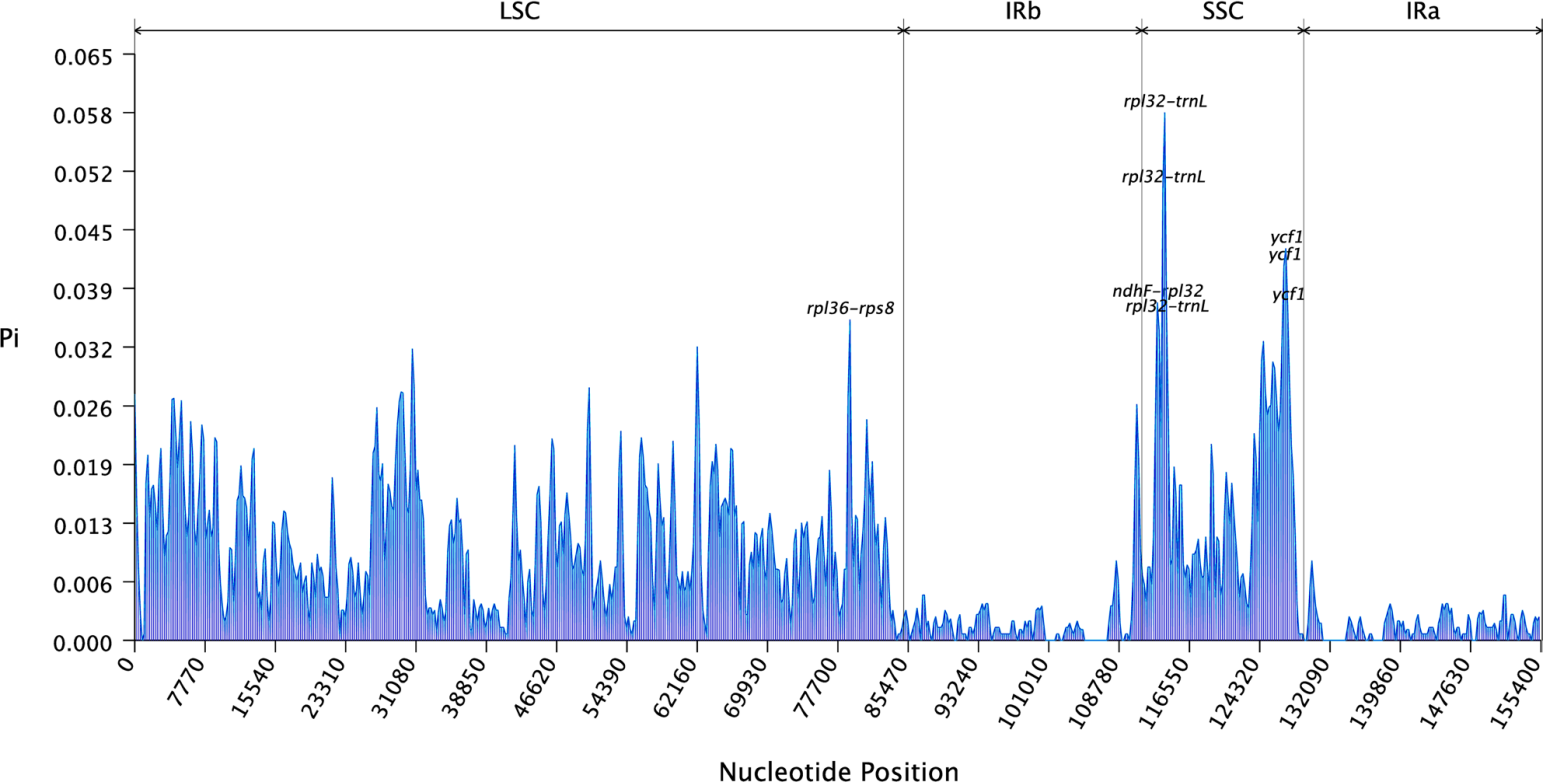
Sliding window analysis of nucleotide diversity (*π*) in *Isatis* chloroplast genomes. The analysis was conducted with a window size of 400 bp and a step size of 200 bp. The x-axis represents the position in the chloroplast genome, and the y-axis shows the nucleotide diversity (*π*) value per window. Peak regions indicate highly variable loci. Key regions with high *π* values are labeled

Nucleotide diversity analysis was conducted on 132 genes within the chloroplast genomes of *Isatis* species. As shown in Fig. 6, the average nucleotide variability (*π*) for the genus was 0.0083, ranging from 0.0000 to 0.0650. The *rpl32–trn*L region presented a high *π* value of 0.0582, suggesting its potential as a candidate marker for distinguishing *Isatis* species.

### Codon usage characteristics of *Isatis* chloroplast genomes

**Fig. 7:**
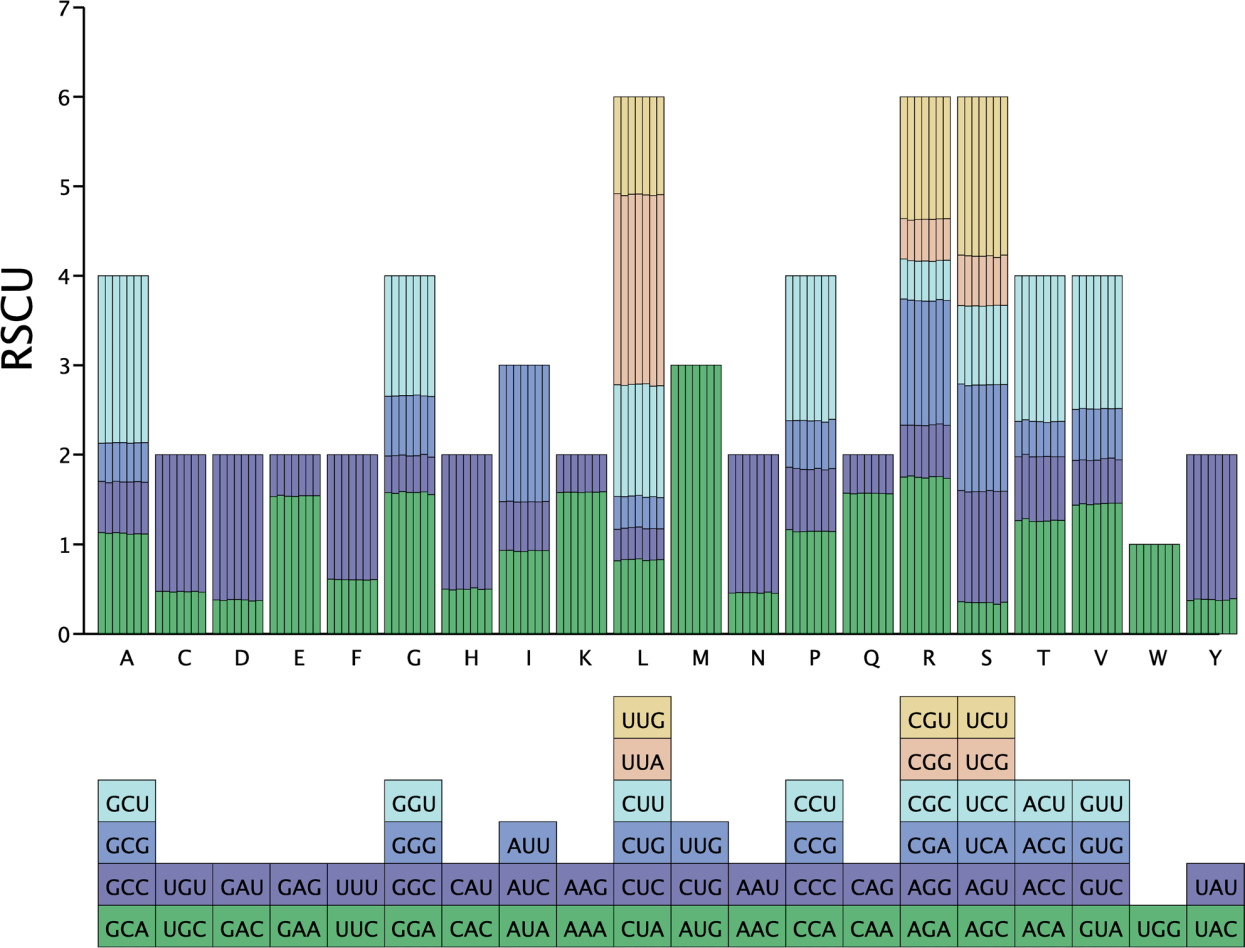
Codon usage bias in *Isatis* chloroplast protein-coding genes. The RSCU values for all 64 codons are shown. Codon families are color-coded by the encoded amino acid

Codon distribution analysis revealed amino acid frequencies ranging from 1.63% (rare) to 9.38% (abundant). Three residues dominated the codon composition: leucine (Leu, 9.38%) preferentially used UUA, arginine (Arg) favoured AGA, and serine (Ser) predominantly employed UCU. Among the 20 amino acids, methionine (Met) and tryptophan (Trp) exclusively relied on the AUG and UGG codons, whereas the other amino acids presented 2–5 synonymous variants. Four-codon amino acids (Ala, Gly, Pro, Thr, and Val) showed distinct preferences for GCU, GGA, CCU, ACU, and GUA, respectively. Among its three synonymous options, isoleucine (Ile) demonstrated AUU dominance. Analysis of 61 codons revealed 35 with RSCU > 1 and 26 with RSCU < 1, indicating codon-ending preferences: 22.95% A/T, 50.82% C/G, and 26.23% U (Fig. 7).

### Evolutionary constraints on chloroplast genome evolution

The Ka/Ks ratio (nonsynonymous/synonymous substitution rates) serves as an evolutionary pressure indicator, where ratios > 1 and < 1 indicate positive and purifying selection, respectively. All the examined *Isatis* species presented Ka/Ks values less than 0.4 across chloroplast genes (Table 4), revealing two predominant evolutionary patterns: (1) the universal presence of purifying selection and (2) strong functional conservation during angiosperm evolution. Notably, a few exceptions were observed; for instance, the rpoA gene exhibited the highest Ka/Ks value (2.36) in the comparison between *I. costata* and *I. minima*. This conservation pattern suggests the stringent elimination of deleterious mutations through natural selection.

**Table 4:** Genes under positive selection and genome-wide selection pressure in pairwise comparisons of *Isatis* species

Species pair	Gene under positive selection	Ka/Ks (gene)	Genome-wide Ka	Genome-wide Ks	Genome-wide Ka/Ks
*I. costata* vs. *I. minima*	*rpoA*	2.3550	0.1154	0.6649	0.1736
*I. multicaulis* vs. *I. gymnocarpa*	*cemA*	1.6544	0.2539	1.0977	0.2313
*I. indigotica* vs. *I. minima*	*ccsA*	1.4044	0.1046	0.4434	0.2360
*I. tinctoria* vs. *I. costata*	*accD*	1.4025	0.1113	0.5572	0.1998
*I. violascens* vs. *I. costata*	*rps16*	1.3802	0.1863	0.8058	0.2312
*I. violascens* vs. *I. gymnocarpa*	*ndhG*	1.2952	0.0905	0.3475	0.2605
*I. indigotica* vs. *I. costata*	*ccsA*	1.1763	0.1024	0.4755	0.2154
*I. multicaulis* vs. *I. violascens*	*cemA*	1.1744	0.2156	0.9341	0.2308
*I. multicaulis* vs. *I. tinctoria*	NA	NA	0.1791	0.8219	0.2179
*I. multicaulis* vs. *I. indigotica*	NA	NA	0.1686	0.7765	0.2172
*I. multicaulis* vs. *I. costata*	NA	NA	0.1815	0.8517	0.2132
*I. multicaulis* vs. *I. minima*	NA	NA	0.1595	0.7876	0.2025
*I. violascens* vs. *I. tinctoria*	NA	NA	0.1551	0.6435	0.2410
*I. violascens* vs. *I. indigotica*	NA	NA	0.1462	0.6164	0.2371
*I. violascens* vs. *I. minima*	NA	NA	0.1863	0.8058	0.2312
*I. tinctoria* vs. *I. indigotica*	NA	NA	0.1551	0.6435	0.2410
*I. tinctoria* vs. *I. minima*	NA	NA	0.1139	0.5272	0.2161
*I. tinctoria* vs. *I. gymnocarpa*	NA	NA	0.1809	0.9258	0.1954
*I. indigotica* vs. *I. gymnocarpa*	NA	NA	0.1705	0.8482	0.2010
*I. costata* vs. *I. gymnocarpa*	NA	NA	0.2205	0.9750	0.2262
*I. minima* vs. *I. gymnocarpa*	NA	NA	0.1997	0.8485	0.2353

The genome-wide values are averages across all protein-coding genes. NA indicates that no gene with a Ka/Ks > 1 was detected

### Phylogenetic relationships based on chloroplast genome sequences

**Fig. 8:**
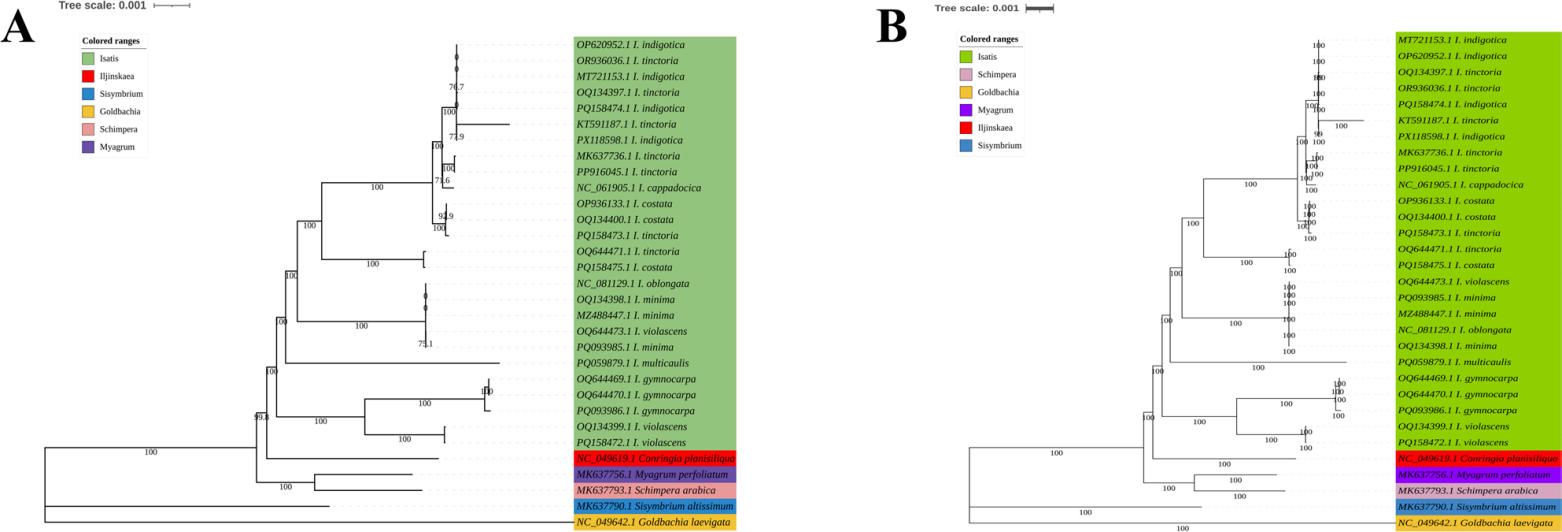
Phylogenomic reconstruction of *Isatis* and the tribe Isatideae using whole chloroplast genomes. Numbers at branches indicate ML bootstrap values (left) and BI posterior probabilities (right). Sections are color-coded, and *Sisymbrium altissimum* was used as the outgroup. GenBank accession numbers are shown. For full taxonomic details (genus and tribe), see Table S1

To elucidate the phylogenetic position of the genus *Isatis* within the Brassicodae supertribe and the relationships among its species, we constructed phylogenetic trees from chloroplast genome sequences using both ML and BI methods (Fig. 8A, B). The topologies of the ML and BI trees were highly congruent, with only minor differences in the placement of a few conspecific accessions. The phylogenetic analysis clearly resolved several major clades among the studied Brassicodae species. First, the tribe Isatideae was robustly supported as a monophyletic group with 100% bootstrap support. This tribe comprised a core *Isatis* clade, along with *Myagrum perfoliatum*, *Schimpera arabica*, and *Iljinskaea planisiliqua*, which together formed a highly supported (100% bootstrap support (BS)) monophyletic clade, thereby clarifying their systematic positions within Isatideae. The genus *Isatis* itself was also strongly supported as monophyletic (100% BS), indicating that the species studied here share a most recent common ancestor. Notably, *I. gymnocarpa* and *I. multicaulis*, which were previously transferred into *Isatis*, were robustly nested within the *Isatis* clade, confirming their current taxonomic placement within this genus. As expected, *Sisymbrium altissimum* from the tribe Sisymbrieae, used as the outgroup, was positioned outside the Isatideae clade. Furthermore, *Goldbachia laevigata*, representing another tribe within the Brassicodae supertribe, was clearly separated from the core clade (comprising Isatideae and Sisymbrieae). This further validates the reliability of our phylogenetic framework and provides a clear context for the circumscription of the Isatideae.

Within *Isatis*, the BI tree confirmed the same complex interspecific relationships and phylogenetic structures as revealed by the ML analysis. The seven core species self-determined in this study and verified by morphology (*I. indigotica*, *I. tinctoria*, *I. costata*, *I. minima*, *I. multicaulis*, *I. gymnocarpa*, and *I. violascens*) were confirmed in their phylogenetic positions with high support, showing clear differentiation. Specifically, the self-determined sequences of *I. violascens* and *I. gymnocarpa* each formed independent, fully supported (100% BS) clades, affirming their status as distinct species entities. The self-determined *I. multicaulis* formed a distinct branch, indicating its unique genetic background. However, upon the inclusion of congeneric sequences from public databases (NCBI), clustering patterns that were inconsistent with their species labels were observed. For instance, the self-determined *I. minima* clustered in a clade with some NCBI sequences labelled as *I. minima*, as well as NCBI sequences labelled as *I. violascens* and *I. oblongata*. The self-determined *I. costata* clustered with one NCBI sequence labelled as *I. tinctoria*, whereas the self-determined *I. tinctoria* clustered with two NCBI sequences labelled as *I. costata*. The self-determined *I. indigotica* clustered into a large, highly supported clade with all NCBI-derived *I. indigotica* sequences, a subset of NCBI-derived *I. tinctoria* sequences, and *I. cappadocica*. These clustering results indicate that the genetic boundaries within *Isatis* for certain species (e.g., *I. costata*, *I. tinctoria*, and *I. indigotica*) may be more complex than those defined by traditional morphological taxonomy. Furthermore, an interesting phylogenetic structure was observed: *I. minima* and *I. violascens*, which are highly similar in morphology and distribution, did not form a direct sister clade. Instead, their respective clades were separated by those of *I. gymnocarpa* and *I. multicaulis*. This may be attributed to their similar habitats, which requires further investigation.

### The *rpl*32–*trn*L intergenic spacer effectively discriminates most *Isatis* species

To evaluate the discriminatory power of the highly variable “*rpl32–trn*L” intergenic spacer—identified through chloroplast genome screening—at the species level, we sequenced 40 samples representing seven core species. Two outgroups (*Myagrum perfoliatum* and *Sisymbrium orientale*) were included, and phylogenetic trees were reconstructed using both ML and NJ methods.

**Fig. 9:**
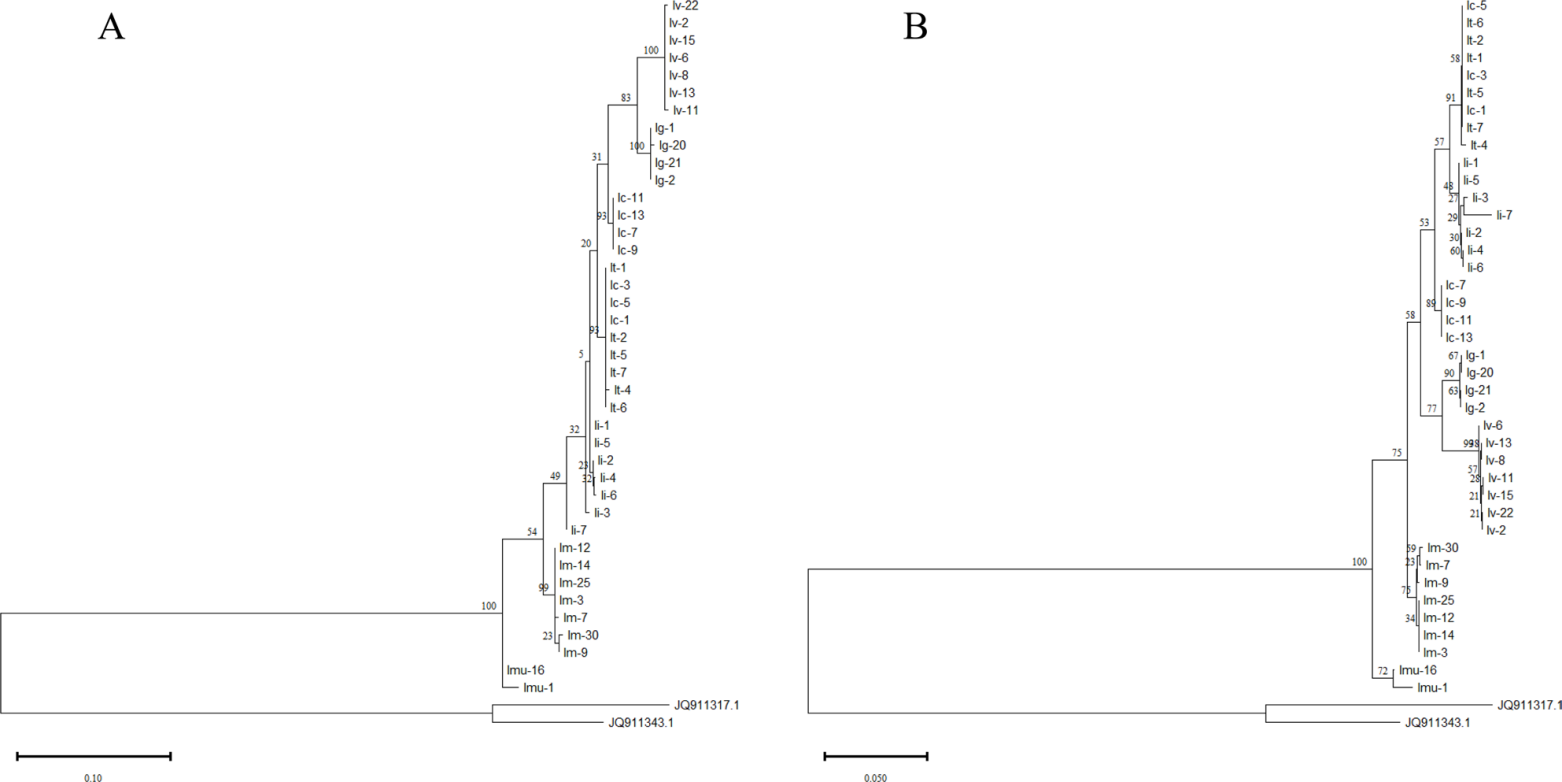
Phylogenetic analysis based on the *rpl32–trn*L intergenic spacer. The analysis included 40 *Isatis* accessions and two outgroup species (*Myagrum perfoliatum* and *Sisymbrium orientale*). Trees were reconstructed using (**A**) ML and (**B**) NJ methods. Numbers at branches represent bootstrap support values from ML (left) and NJ (right) analyses

The ML and NJ trees revealed largely congruent topologies regarding species-level relationships (Fig. 9A, B). The marker successfully delineated several species into highly supported monophyletic clades, including *I. violascens* (ML-BS = 100%), *I. gymnocarpa* (ML-BS = 100%), *I. minima* (ML-BS = 99%), *I. multicaulis* (ML-BS = 100%), and two distinct, highly supported lineages within the *I. costata* complex (both ML-BS = 93%). These results confirm that the “*rpl32–trn*L” spacer is highly polymorphic and possesses strong discriminatory power among most *Isatis* species.

The analysis also revealed complex relationships. Although all samples of *I. indigotica* formed a monophyletic cluster in both trees, internal branches received low support (BS < 60%). Furthermore, one lineage of the *I. costata* complex (comprising Ic-1, Ic-3, and Ic-5) was phylogenetically indistinguishable from all samples of *I. tinctoria*. In all analyses, the two outgroups were positioned at the outermost branches.

## Discussion

### Comparative analysis of chloroplast genomes in *Isatis*

Chloroplasts, the organelles responsible for photosynthesis and biosynthesis in photosynthetic organisms, provide organic compounds and energy essential for their life processes [34]. These semiautonomous organelles possess a closed circular double-stranded DNA structure and a chloroplast genome independent of the nuclear genome [35]. Characterized by structural stability, sequence conservation, abundant variable sites in intergenic regions, and a relatively slow molecular evolutionary rate, chloroplast genomes are widely utilized in studies of phylogenetic relationships, systematic evolution, and genetic diversity [36]. The chloroplast genomes of more than a thousand plant species have been sequenced to date. Among these, traditional Chinese medicinal (TCM) plants are being increasingly characterized, constituting a valuable resource for phylogenetic and evolutionary studies. The genomic data from TCM species such as *Cistanche deserticola* Y.C. Ma [37], *Platycodon grandiflorus* (Jacq.) A. DC [38]. , *Polygala tenuifolia* Willd [39]. , and *Clematis henryi* Oliv [40]. provide critical references, thereby facilitating future research on a broader range of species. These sequencing results have played a significant role in elucidating plant phylogenetic positions, species identification, and evolutionary analyses [41]. Investigating codon usage patterns in plant chloroplast genomes provides valuable data for enhancing the efficiency of gene expression vector construction, exploring species evolutionary relationships, understanding the molecular mechanisms of biological adaptation to the environment, and improving crop germplasm [9]. By constructing the chloroplast genomes of *Isatis*, sequence variations among different germplasms can be identified, enabling more precise species discrimination within the genus and resolving taxonomic controversies. Furthermore, this approach provides molecular markers and genetic resources for the genetic breeding of *Isatis* species, accelerating the process of varietal improvement.

Comparative analysis of the *Isatis* chloroplast genomes revealed a conserved quadripartite architecture with limited IR variation (< 2% length difference), in contrast to the relatively high divergence in single-copy regions (LSC/SSC: 5.8–7.4% boundary shifts). Sequence variation exhibited spatial gradients: SSC (12.3 single-nucleotide polymorphisms (SNPs)/kb) > LSC (8.9) > IRs (1.7) and functional partitioning, with noncoding regions showing 3.2× greater polymorphism than coding sequences (*P* < 0.001). Molecular signatures, including codon usage bias (RSCU = 1.15–1.82) and SSR distribution (42–47 loci/genome), followed core eudicot patterns. These findings collectively support a dual evolutionary model, namely, strong functional constraints maintaining IR/coding region stability versus neutral evolution driving diversification in single-copy/noncoding regions, which is consistent with genus-wide evolutionary trajectories.

### Analysis of codon usage in the chloroplast genomes of *Isatis*

Codon usage in chloroplast genomes refers to the triplet nucleotide sequences encoding proteins within chloroplast DNA [42]. Although these codons are largely similar to those in the nuclear genome, some differences exist [43]. Codon usage can be analysed for preferences, selection pressures, and polymorphic sites [44]. In-depth studies on codon usage patterns and their influencing factors in plant chloroplast genomes provide a theoretical basis for vector selection in genetic engineering and gene expression [45]. Such analyses also aid in predicting the expression of unknown genes or identifying potential functional genes, which is highly important for studies on species evolution and genetics [46].

The chloroplast genome of *Isatis* exhibits adaptive codon usage bias characterized by A/U-ending preference (RSCU: 1.15–1.82) and low GC content at the third codon position (< 42%) [47], which aligns with conserved translational optimization strategies in higher plants such as Trigonella [48], Glehnia [49], and Sorghum [50]. This A/T-rich codon bias is correlated with elevated gene expression levels [10], reflecting genomic adaptation to environmental and evolutionary pressures [44]. Structural analysis revealed a conserved quadripartite architecture, with IRs showing < 2% length variation, in contrast with 5.8–7.4% boundary shifts in single-copy regions (LSC/SSC). These findings are consistent with the dual evolutionary model of functional constraint and neutral divergence.

Ka/Ks analysis across seven *Isatis* species revealed stringent purifying selection (mean < 0.3) in core photosynthesis-related genes, including photosystem components (*psbD*–*Z*), the cytochrome b/f complex (*petB*–*N*), and ATP synthase subunits (*atpB*–*I*), with near-zero substitution rates (*P* < 0.001). Conversely, uncharacterized genes (*ycf1*–*2*: 0.68–0.89) and protease-related loci (*clpP*, *matK*: 0.51–0.63) presented moderate positive selection signals. This functional dichotomy underscores stronger codon bias in highly expressed metabolic genes than adaptive flexibility in accessory genes, mirroring eudicot chloroplast evolutionary trajectories [44, 51].

### Phylogenetic implications and taxonomic challenges

By constructing the first comprehensive phylogeny of *Isatis* based on complete chloroplast genomes, this study provides an independent and robust assessment of the evolutionary history of the genus, addressing several longstanding taxonomic questions. Our analysis not only confirms the monophyly of the tribe Isatideae and the genus *Isatis* with maximum support (100% BS) but also corroborates the phylogenetic placement of the tribe within the Brassicodae supertribe, aligning with and reinforcing previous findings from multilocus nuclear data [13]. This establishes a reliable evolutionary context for resolving relationships within this complex genus.

A key finding concerns the recent reclassification of *I. gymnocarpa* and *I. multicaulis* into *Isatis*. Their stable nesting within the core *Isatis* clade in our chloroplast genome phylogeny provides compelling independent evidence that validates these genus transfers, which were initially proposed based on morphological and limited nuclear evidence [14, 15, 52]. However, the infrageneric relationships revealed significant complexity. Although the seven morphologically verified core species formed well-supported, distinct clades, the inclusion of publicly available sequences revealed substantial discrepancies. We observed pronounced incongruence between molecular clustering and species labels for critical taxa such as *I. costata*, *I. tinctoria*, and *I. indigotica*. This pattern most parsimoniously points to widespread misidentification in public databases, an issue exacerbated by the morphological similarity within the genus. Although incomplete lineage sorting could contribute, the scale of the inconsistency strongly suggests that the current morphology-based species boundaries for these taxa are untenable and require critical re-evaluation.

The resolution offered by the complete chloroplast genome also enables the identification of highly variable regions suitable for DNA barcoding. Our discovery of the hypervariable *rpl*32–*trn*L region (*π* = 0.0582) aligns with and extends prior work, which identified the homologous region as an effective mini-barcode for distinguishing *I. indigotica* and *I. tinctoria* [9]. To empirically validate its discriminatory power, we conducted a phylogenetic analysis of 40 samples across the seven core species using this specific region. This analysis confirmed its effectiveness as a mini-barcode, robustly resolving most species into highly supported monophyletic clades. However, it also revealed a more complex pattern within *I. costata*, which was split into two highly supported lineages, one of which was inseparable from *I. tinctoria*. This incongruence with the species boundaries suggested by the chloroplast genome phylogeny points to a complex evolutionary history, potentially involving hybridization or chloroplast capture, that merits future investigation. This convergence of evidence underscores the dual utility of the *rpl*32–*trn*L region as both a practical identification tool and a probe for uncovering deeper evolutionary dynamics within the genus. Furthermore, our phylogeny offers insights into evolutionary processes beyond simple relationships. The observation that the morphologically similar *I. minima* and *I. violascens* do not form sister clades is particularly instructive. Given their shared dune habitat, this phylogenetic separation strongly implies that their phenotypic similarity is a result of convergent evolution driven by adaptation to analogous ecological pressures rather than a shared recent ancestry.

## Conclusion

In this study, a multifaceted analysis of the chloroplast genomes in the genus *Isatis* was conducted through comparative genomics, codon usage bias, and phylogenetic analyses. The results demonstrate that the *Isatis* chloroplast genome adheres to the typical evolutionary patterns of core eudicots, which exhibit spatial heterogeneity in structural variation (highly conserved IR regions vs. significantly divergent single-copy regions) and sequence polymorphism (noncoding > coding regions), driven collectively by functional constraints and neutral evolution. Codon usage preference analysis further revealed an adaptive A/U-ending bias and strong purifying selection on core photosynthesis-related genes, collectively underscoring the evolutionary conservation of the genome. Phylogenomic analyses strongly supported the monophyly of the tribe Isatideae and the genus *Isatis* and confirmed the taxonomic placement of *I. gymnocarpa* and *I. multicaulis* within the genus. However, significant species misidentification in public databases was detected, particularly for critical taxa such as *I. costata*, *I. tinctoria*, and *I. indigotica*, indicating that current morphology-based species boundaries may not reflect their genetic delimitations. The *rpl*32–*trn*L marker further revealed the complex nature of the *I. costata* group, where some individuals were inseparable from *I. tinctoria*, suggesting potential hybridization or chloroplast capture. Furthermore, the morphologically similar *I. minima* and *I. violascens* were not sister species, implying that their similarity likely resulted from convergent evolution in analogous habitats rather than recent shared ancestry. This study provides crucial genomic resources and a theoretical foundation for species identification, systematic evolution, and genetic breeding in *Isatis*.

## Supplementary Information

Supplementary Material 1.

Supplementary Material 2.

Supplementary Material 3.

## Data Availability

The chloroplast genomes of *Isatis* have been uploaded to NCBI (GenBank accession numbers: *I. indigotica* (PQ158474), *I. costata* (PQ158475), *I. tinctoria* (PQ158473), *I. violascens* (PQ158472), *I. minima* (PQ093985), *I. gymnocarpa* (PQ093986), and *I. multicaulis* (PQ059879)). The datasets supporting the findings of this study, including all supplementary figures and tables, are available in the Figshare repository at 10.6084/m9.figshare.30315475.
