# Supplementary material for: Insights into the chloroplast genome diversity of the genus Isatis in China
Source: BMC Plant Biol. 2026 Jan 26;26:336. doi: 10.1186/s12870-026-08240-3 (PMC12914916; doi:10.1186/s12870-026-08240-3)
Supplement: Supplementary file 1 — Supplementary Material 1. [file 12870_2026_8240_MOESM1_ESM.docx]

**List of Abbreviations**

| Abbreviation | Full Form |
| --- | --- |
| BI | Bayesian inference |
| BS | Bootstrap support |
| CDSs | Coding sequences |
| DNA | Deoxyribonucleic acid |
| GC | Guanine-cytosine |
| IGSs | Intergenic spacers |
| iTOL | Interactive Tree of Life |
| IR | Inverted repeat |
| Ka/Ks | Nonsynonymous/synonymous substitution rates |
| LSC | Large single-copy |
| MCMC | Markov chain Monte Carlo |
| ML | Maximum likelihood |
| NCBI | National Center for Biotechnology Information |
| NJ | Neighbour-Joining |
| ORF | Open reading frame |
| PCGs | Protein-coding genes |
| PCR | Polymerase chain reaction |
| *π* | Nucleotide diversity |
| RSCU | Relative synonymous codon usage |
| RNA | Ribonucleic acid |
| SNPs | Single-nucleotide polymorphisms |
| SSRs | Simple sequence repeats |
| SSC | Small single-copy |
| TCM | Traditional Chinese medicinal |
