# Supplementary material for: Insights into the chloroplast genome diversity of the genus Isatis in China
Source: BMC Plant Biol. 2026 Jan 26;26:336. doi: 10.1186/s12870-026-08240-3 (PMC12914916; doi:10.1186/s12870-026-08240-3)
Supplement: Supplementary file 3 — Supplementary Material 3. [file 12870_2026_8240_MOESM3_ESM.docx]

**Table S1. Summary of samples used in the phylogenetic analysis**

| Number | Species | Accession No. | Status | Long./Lat. | Sample ID | Reference | Tribe |
| --- | --- | --- | --- | --- | --- | --- | --- |
| **1** | ***Isatis indigotica*** | **PQ158474** | **Cultivated** | **115.07E/29.92N** | **Ii-1** | **This study** | **Isatideae** |
| **2** | ***Isatis costata*** | **PQ158475** | **Wild** | **85.03E/46.87N** | **Ic-13** | **This study** | **Isatideae** |
| **3** | ***Isatis*** ***tinctoria*** | **PQ158473** | **Wild** | **86.93E/48.88N** | **It-1** | **This study** | **Isatideae** |
| **4** | ***Isatis violascens*** | **PQ158472** | **Wild** | **88.38E/44.96N** | **Iv-6** | **This study** | **Isatideae** |
| **5** | ***Isatis minima*** | **PQ093985** | **Wild** | **87.42E/45.64N** | **Im-25** | **This study** | **Isatideae** |
| **6** | ***Isatis*** ***gymnocarpa*** | **PQ093986** | **Wild** | **87.80E/44.06N** | **Ig-1** | **This study** | **Isatideae** |
| **7** | ***Isatis multicaulis*** | **PQ059879** | **Wild** | **88.76E/44.36N** | **Imu-16** | **This study** | **Isatideae** |
| 8 | *Isatis tinctoria* | PP916045.1 | Unknown | N/A | Not provided | Direct submission | Isatideae |
| 9 | *Isatis tinctoria* | OR936036.1 | Unknown | N/A | Not provided | Direct submission | Isatideae |
| 10 | *Isatis tinctoria* | OQ644471.1 | Unknown | N/A | Not provided | Direct submission | Isatideae |
| 11 | *Isatis tinctoria* | KT591187.1 | Unknown | N/A | Not provided | Direct submission | Isatideae |
| 12 | *Isatis tinctoria* | OQ134397.1 | Unknown | N/A | Not provided | Direct submission | Isatideae |
| 13 | *Isatis tinctoria* | MK637736.1 | Unknown | N/A | Not provided | Direct submission | Isatideae |
| 14 | *Isatis indigotica* | PX118598.1 | Unknown | N/A | Not provided | Direct submission | Isatideae |
| 15 | *Isatis indigotica* | OP620952.1 | Wild | 118.85E/32.04N | Not provided | Su et al., 2023 | Isatideae |
| 16 | *Isatis indigotica* | MT721153.1 | Unknown | N/A | Not provided | Direct submission | Isatideae |
| 17 | *Isatis costata* | OP936133.1 | Unknown | N/A | Not provided | Direct submission | Isatideae |
| 18 | *Isatis costata* | OQ134400.1 | Unknown | N/A | Not provided | Direct submission | Isatideae |
| 19 | *Isatis violascens* | OQ644473.1 | Unknown | N/A | Not provided | Direct submission | Isatideae |
| 20 | *Isatis violascens* | OQ134399.1 | Unknown | N/A | Not provided | Direct submission | Isatideae |
| 21 | *Isatis minima* | MZ488447.1 | Unknown | 87.56E/43.82N | IBSC 0812950 | Song et al., 2022 | Isatideae |
| 22 | *Isatis minima* | OQ134398.1 | Unknown | N/A | Not provided | Direct submission | Isatideae |
| 23 | *Isatis gymnocarpa* | OQ644470.1 | Unknown | N/A | Not provided | Direct submission | Isatideae |
| 24 | *Isatis gymnocarpa* | OQ644469.1 | Unknown | N/A | Not provided | Direct submission | Isatideae |
| 25 | *Isatis cappadocica* | NC_061905.1 | Unknown | 121.59E/ 31.19N | IsCAP001 | Fang et al., 2022 | Isatideae |
| 26 | *Isatis oblongata* | NC_081129.1 | Unknown | N/A | Not provided | Direct submission | Isatideae |
| 27 | *Myagrum perfoliatum* | MK637756.1 | Unknown | N/A | Not provided | Direct submission | Isatideae |
| 28 | *Schimpera arabica* | MK637793.1 | Unknown | N/A | Not provided | Direct submission | Isatideae |
| 29 | *Conringia planisiliqua* | NC_049619.1 | Unknown | N/A | Not provided | Direct submission | Isatideae |
| 30 | *Sisymbrium altissimum* | MK637790.1 | Unknown | N/A | Not provided | Direct submission | Sisymbrieae |
| 31 | *Goldbachia laevigata* | NC_049642.1 | Unknown | N/A | Not provided | Direct submission | Calepineae |

*Note:* GenBank accession numbers for the 31 accessions used for phylogenetic tree construction in this study. Newly sequenced chloroplast genomes in this study are shown in bold.

**Table S2. Sample information for the phylogenetic analysis based on intergenic spacer regions**

| Number | Locus | Sample ID | Species | Geographic Location | Long./Lat. | Status |
| --- | --- | --- | --- | --- | --- | --- |
| 1 | *rpl32–trn*L | Ic-1 | *Isatis costata* | Buerjin, Xinjiang | 87.24E/48.43N | Wild |
| 2 | *rpl32–trn*L | Ic-3 | *Isatis costata* | Buerjin, Xinjiang | 87.29E/48.44N | Wild |
| 3 | *rpl32–trn*L | Ic-5 | *Isatis costata* | Buerjin, Xinjiang | 87.36E/48.52N | Wild |
| 4 | *rpl32–trn*L | Ic-7 | *Isatis costata* | Habahe, Xinjiang | 86.56E/48.31N | Wild |
| 5 | *rpl32–trn*L | Ic-9 | *Isatis costata* | Habahe, Xinjiang | 86.58E/48.30N | Wild |
| 6 | *rpl32–trn*L | Ic-11 | *Isatis costata* | Jimunai, Xinjiang | 86.12E/47.18N | Wild |
| 7 | *rpl32–trn*L | Ic-13 | *Isatis costata* | Hebukesaier, Xinjiang | 85.03E/46.87N | Wild |
| 8 | *rpl32–trn*L | Iv-2 | *Isatis violascens* | Fukang, Xinjiang | 88.14E/44.37N | Wild |
| 9 | *rpl32–trn*L | Iv-6 | *Isatis violascens* | Fukang, Xinjiang | 88.38E/44.96N | Wild |
| 10 | *rpl32–trn*L | Iv-8 | *Isatis violascens* | Fukang, Xinjiang | 88.96E/44.95N | Wild |
| 11 | *rpl32–trn*L | Iv-11 | *Isatis violascens* | Fuyun, Xinjiang | 89.44E/45.42N | Wild |
| 12 | *rpl32–trn*L | Iv-13 | *Isatis violascens* | Jimusaer, Xinjiang | 88.85E/44.68N | Wild |
| 13 | *rpl32–trn*L | Iv-15 | *Isatis violascens* | Jimusaer, Xinjiang | 88.76E/44.36N | Wild |
| 14 | *rpl32–trn*L | Iv-22 | *Isatis violascens* | Fuhai, Xinjiang | 87.42E/45.64N | Wild |
| 15 | *rpl32–trn*L | Im-3 | *Isatis minima* | Fukang, Xinjiang | 88.14E/44.37N | Wild |
| 16 | *rpl32–trn*L | Im-7 | *Isatis minima* | Fukang, Xinjiang | 88.38E/44.96N | Wild |
| 17 | *rpl32–trn*L | Im-9 | *Isatis minima* | Jimusaer, Xinjiang | 88.96E/44.95N | Wild |
| 18 | *rpl32–trn*L | Im-12 | *Isatis minima* | Fuyun, Xinjiang | 89.44E/45.42N | Wild |
| 19 | *rpl32–trn*L | Im-14 | *Isatis minima* | Jimusaer, Xinjiang | 88.85E/44.68N | Wild |
| 20 | *rpl32–trn*L | Im-25 | *Isatis minima* | Fuyun, Xinjiang | 87.42E/45.64N | Wild |
| 21 | *rpl32–trn*L | Im-30 | *Isatis minima* | Fuyun, Xinjiang | 87.57E/45.52N | Wild |
| 22 | *rpl32–trn*L | It-1 | *Isatis tinctoria* | Habahe, Xinjiang | 86.87E/48.84N | Wild |
| 23 | *rpl32–trn*L | It-2 | *Isatis tinctoria* | Habahe, Xinjiang | 86.87E/48.84N | Wild |
| 24 | *rpl32–trn*L | It-4 | *Isatis tinctoria* | Habahe, Xinjiang | 86.93E/48.88N | Wild |
| 25 | *rpl32–trn*L | It-5 | *Isatis tinctoria* | Habahe, Xinjiang | 86.93E/48.88N | Wild |
| 26 | *rpl32–trn*L | It-6 | *Isatis tinctoria* | Habahe, Xinjiang | 86.93E/48.88N | Wild |
| 27 | *rpl32–trn*L | It-7 | *Isatis tinctoria* | Habahe, Xinjiang | 86.81E/48.97N | Wild |
| 28 | *rpl32–trn*L | Ig-1 | *Isatis gymnocarpa* | Midong, Xinjiang | 87.80E/44.15N | Wild |
| 29 | *rpl32–trn*L | Ig-2 | *Isatis gymnocarpa* | Midong, Xinjiang | 87.80E/44.06N | Wild |
| 30 | *rpl32–trn*L | Ig-20 | *Isatis gymnocarpa* | Fuhai, Xinjiang | 87.54E/46.08N | Wild |
| 31 | *rpl32–trn*L | Ig-21 | *Isatis gymnocarpa* | Fuhai, Xinjiang | 87.54E/46.23N | Wild |
| 32 | *rpl32–trn*L | Imu-1 | *Isatis multicaulis* | Jimusaer, Xinjiang | 88.76E/44.36N | Wild |
| 33 | *rpl32–trn*L | Imu-16 | *Isatis multicaulis* | Jimusaer, Xinjiang | 88.59E/44.45N | Wild |
| 34 | *rpl32–trn*L | Ii-1 | *Isatis indigotica* | Yangxin, Hubei | 115.07E/29.92N | Cultivated |
| 35 | *rpl32–trn*L | Ii-2 | *Isatis indigotica* | Qianxian, Shaanxi | 108.20E/34.50N | Cultivated |
| 36 | *rpl32–trn*L | Ii-3 | *Isatis indigotica* | Jinxiang, Shandong | 116.34E/35.02N | Cultivated |
| 37 | *rpl32–trn*L | Ii-4 | *Isatis indigotica* | Dapu, Guangdong | 116.64E/24.38N | Cultivated |
| 38 | *rpl32–trn*L | Ii-5 | *Isatis indigotica* | Luoding, Guangdong | 111.57E/22.84N | Cultivated |
| 39 | *rpl32–trn*L | Ii-6 | *Isatis indigotica* | Fangcheng, Henan | 112.84E/33.24N | Cultivated |
| 40 | *rpl32–trn*L | Ii-7 | *Isatis indigotica* | Taibai, Shaanxi | 107.37E/34.12N | Cultivated |
